# Model for large-area monolayer coverage of polystyrene nanospheres by spin coating

**DOI:** 10.1038/srep40888

**Published:** 2017-01-19

**Authors:** Abhishek Chandramohan, Nikolai V. Sibirev, Vladimir G. Dubrovskii, Michael C. Petty, Andrew J. Gallant, Dagou A. Zeze

**Affiliations:** 1 Durham University, School of Engineering and Computing Sciences, Durham, DH1 3LE, United Kingdom; 2 St. Petersburg Academic University, St. Petersburg, 194021, Russia; 3 ITMO University, St. Petersburg, 197101, Russia; 4 Ioffe Physical Technical Institute of the Russian Academy of Sciences, St. Petersburg, 194021, Russia

## Abstract

Nanosphere lithography, an inexpensive and high throughput technique capable of producing nanostructure (below 100 nm feature size) arrays, relies on the formation of a monolayer of self-assembled nanospheres, followed by custom-etching to produce nanometre size features on large-area substrates. A theoretical model underpinning the self-ordering process by centrifugation is proposed to describe the interplay between the spin speed and solution concentration. The model describes the deposition of a dense and uniform monolayer by the implicit contribution of gravity, centrifugal force and surface tension, which can be accounted for using only the spin speed and the solid/liquid volume ratio. We demonstrate that the spin recipe for the monolayer formation can be represented as a pathway on a 2D phase plane. The model accounts for the ratio of polystyrene nanospheres (300 nm), water, methanol and surfactant in the solution, crucial for large area uniform and periodic monolayer deposition. The monolayer is exploited to create arrays of nanoscale features using ‘short’ or ‘extended’ reactive ion etching to produce 30–60 nm (diameter) nanodots or 100–200 nm (diameter) nanoholes over the entire substrate, respectively. The nanostructures were subsequently utilized to create master stamps for nanoimprint lithography.

Nanosphere Lithography (NSL), referred to as “Natural Lithography” by Deckman *et al*.^1^ back in 1982 and pioneered by Van Duyne’s group^2^,^3^,^4^,^5^ in the late 1990 s, has come a long way by manifesting itself as a fast, low cost and high throughput nanofabrication method to produce regular arrays of nanostructures^6^. NSL can be divided broadly into three steps: colloidal mask generation, diameter control and lift-off. The first step depends on the quality of the deposition of the monolayer of polystyrene nanospheres (PNs) onto the substrates. There are several methods for the formation of self-organized colloidal-crystal films, e.g. gravity sedimentation^7^, electrophoretic deposition^8^, solvent evaporation^9^, the Langmuir–Blodgett (LB) technique^10^, air–water interfacial floating method^11^ and spin coating^12^,^13^. Secondly, the diameter of the nanospheres in the packed arrays is controlled by dry etching^14^, allowing an additive deposition^15^ step where the deposited material passes through tuned apertures to rest on the substrate. Lastly, the PNs are etched away in a solvent by lift-off, leaving onto the substrate, regular arrays of the material deposited. Nanoparticle arrays produced by this method are often used as surface enhanced Raman scattering substrates^6^ for biological and chemical sensors as well as catalysts for the growth of one-dimensional nanostructures^14^,^15^,^16^,^17^,^18^. Industry has pushed researchers far enough to implement and make the user-friendly spin-coating process viable and able to produce films with controlled thicknesses over large areas. Despite the apparent ease, parameters such as concentration and temperature have a vital impact on the evaporation of the PNs solution. The evaporation rate can be increased by spinning the substrate at higher speeds, albeit the characterization of the samples produced at different spin speeds lead to different coverage, uniformity and packing. Nagayama *et al*.^19^,^20^,^21^ instigated the work on the ordering mechanism of PNs whereas the 3-D colloidal crystal growth was studied by Scriven *et al*.^22^. Emslie *et al*.^23^ laid out the foundation for the theoretical study of nonparticulate films of precise thickness during spin coating. While the principle of the Langmuir-Blodgett thin film technique is well documented in the literature, very little is reported on spin coating of colloidal suspension that correlates the underpinning physics to the spin process involved to improve the packing density and large area surface coverage for batch processing. Recently, Shinde *et al*.^24^ demonstrated a spin coating recipe for PNs for large areas. However, a generic model to help achieve a reproducible, desired coverage and periodicity is still missing. This paper discusses the production of dense 2D self-ordered monolayers of PNs on large-area substrates (2 inch Si wafer) using spin coating. The formulation of the process recipe is based on a model developed to gain a better insight into the correlation between the forces contributing to the various stages of the spin process. The main objective is to determine a suitable composition of the solution of PNs (300 nm) that is sufficient enough to obtain a reproducible and uniform coverage over large-area through a sequential spin-coating method, which has not been discussed in existing literature.

## Results and Discussion

### Boundary conditions

The main forces acting during spin coating are gravity, inertia, surface tension and friction. At a lower spin speed (<1000 RPM), inertia is insignificant and dry friction is less prominent than gravitational force. The latter is independent of the process parameters while centrifugal force is determined by the rotation speed. The role of surface tension is crucially dependent on the amount of solvent and will become dominant for a small volume of solvent. In brief, the analysis of the boundary conditions (detailed in Supplementary Material) shows that the rotation speed and solid/liquid volume ratio (R) are sufficient to characterize the spin coating process that leads to the formation PNs close-packed monolayers over large area substrates.

**Figure 1:**
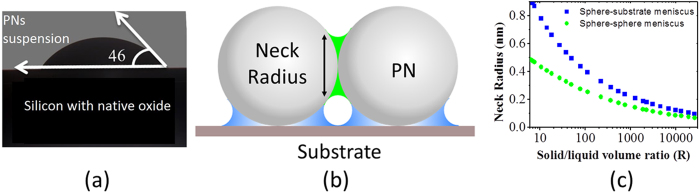
(**a**) Contact angle for a drop of PNs suspension on Si with native oxide (**b**) sphere-sphere (necking radius-green) and sphere-substrate curvature (meniscus-blue) (**c**) meniscus neck radius varying with solid/liquid ratio (R).

Thus, to gain a better insight into the ordering of PNs, only these two process parameters are essential to describe the relative contribution of gravity, centrifugal forces and surface tension (calculated using contact angle, see Fig. 1a). For illustration, the role of R is summarized in Table 1. Surface tension becomes dominant when the curvature of the liquid (Fig. 1b), determined by the solid to liquid ratio (R) of the solid and liquid volume, is negative (Fig. 1c). The curvature of liquid surface turns negative, when the amount of solvent is not sufficient to form a convex hull (Fig. 1b) for all PNs touching each other (Table 1).

The curvature turns negative if R is greater than 3/2, i.e. when the volume of solid spheres and solvent is less than that of the convex hull of all PNs. Hence, the transition point (R_*c*_) complies with (equation 1):


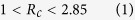


For an initial arbitrary configuration of PNs, we cannot precisely determine R_*c*_ when the solvent volume is not enough to form a convex hull around the PNs. Yet, it must lie between 0.9 and 2.9, see Table 1. R_*c*_ = 1.5 was used to estimate our hexagonal lattice. For a high R, the droplet is uniformly divided into several menisci, where each droplet is wetting only two spheres or a sphere and the substrate. This situation appears when meniscus diameter is less than PNs radius and are of two types – sphere-sphere and sphere-substrate (Fig. 1b).

### Model

**Figure 2:**
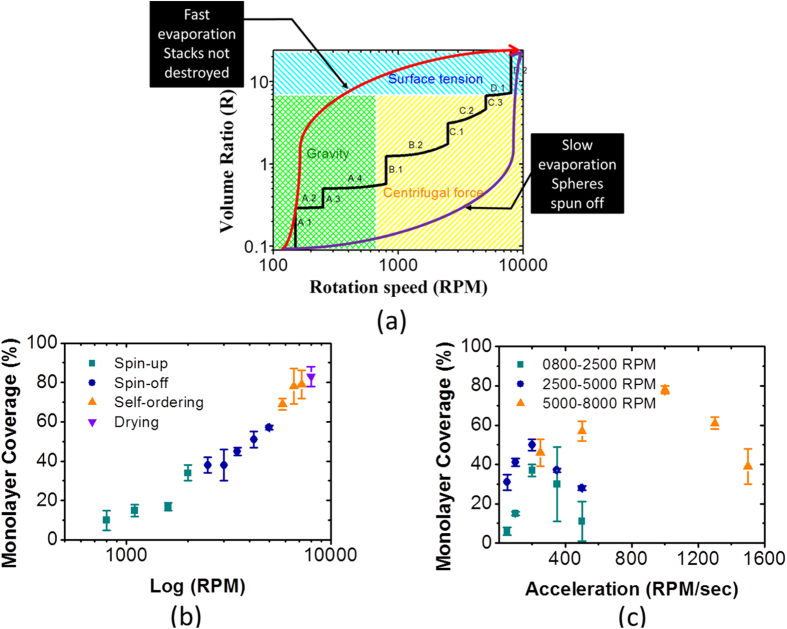
Relationship between (**a**) rotation speed and solid/liquid volume ratio (R) (**b**) monolayer coverage and spin speed (log) (**c**) monolayer coverage and acceleration.

These boundary conditions allow us to consider our process steps as a “process phase space” (or pathway) depicted in Fig. 2a and Table 2. The bottom left corner in Fig. 2a represents the initial diluted solution when the rotation speed is zero, while the top right corner shows that most of the solvent has evaporated at maximum rotation speed. The vertical parts of the pathway correspond to the various stages of the recipe at a constant speed. The inclined concave parts illustrate the transition from one stage to the next. The entire plane can be divided into three main sectors, where gravity, centrifugal force and surface tension dominates, respectively.

For a high value of R (when most of the solvent is evaporated and the remnant is covered by PNs), surface tension causes PNs to stick to each other and to immobilize on the substrate. In contrast, centrifugal force dominates at a higher rotation speed and relatively low R whereas the gravity controlled regime lies in the bottom left corner of the process ‘phase space’. The pathway can pass through all three forces or only through gravity and surface tension. In the latter case, the stacks of spheres cannot be destroyed due to a gravitational force and the surface tension that glues the PNs together onto the surface. If the pathway stays too long in centrifugal force dominated area, inertia can throw away more PNs than necessary, resulting in an insufficient number of PNs to form a dense monolayer. Hence, the path must go through the area dominated by centrifugal force to achieve a uniform close-packed PNs array. Figure 2a shows that this process can be divided into four consecutive key phases with sub-phases: *spin*-*up (A.1*–*4), spin*-*off (B.1*–*2), self*-*ordering (C.1*–*2) and drying (D.1*–*2*). The packing improves as the pathway is systematically followed leading to a monolayer coverage up to 90% (Fig. 2b). In addition, Fig. 2c clearly indicates that the monolayer coverage is strongly affected by acceleration between respective stages. Thus, a careful consideration is needed to construct a suitable pathway.

**Figure 3:**
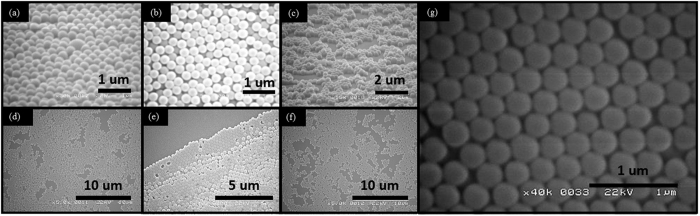
SEM images of PNs in sequential stages showing (**a**) (*A.2*) aggregation (**b**) (*A.4*) flattening (**c**) (*B.2*)) monolayer generation (**d**) (*B.1*) coverage improvement (**e**) (*C.1*) multilayers at edge (**f**) (*C.3*) occupation of void spaces (**g**) (*D.2*) hexagonal packing.

The full process in Table 2 where the effects at each stage are illustrated by scanning electron micrographs (SEM) shown in Fig. 3. In the first phase A.1–4, the solution spreads over the substrate to ensure a uniform solution coverage (Fig. 3b). In phase B.1–2, the inertial force is strong enough to roll them over in an attempt to break PNs lumps (Fig. 3c) into a disordered monolayer and partial substrate coverage (Fig. 3d). During phase C.1–3, PNs above the bottom layer cannot be thrown out but instead roll over to the bottom layer in empty spaces or fall off the substrate. Further evaporation of the solvent pulls the nanospheres together and pushes them into a closed-hexagonal packing as shown in Fig. 3e,f.

The duration, rotation speed and acceleration rate of these stages are determined by a balance between centrifugal forces and surface tensions. On one hand, the rotation speed and time should be large enough to disperse the PNs across the full sample from the center to the edges. On the other, the evaporation rate must be small enough to avoid a breakdown of the droplet into several menisci, which can hinder close packing. The optimal parameters identified in current work are displayed in Table 2 and illustrated in Fig. 2a. In the last phase D.2, unlike the previous stage, the solvent droplet breaks into several sufficiently small menisci that glue the hexagonal closely packed PN to the substrate. However, the variations observed in the diameter of the PNs can lead to irregularities and domains in the packing by disrupting the order and resulting into the stacking of spheres. SEM investigation showed that 95% of the nanospheres had diameters in the range of 280–320 nm where as the remaining 5% may be as large as 4 times the expected size.

### Nanosphere Lithography

**Figure 4:**
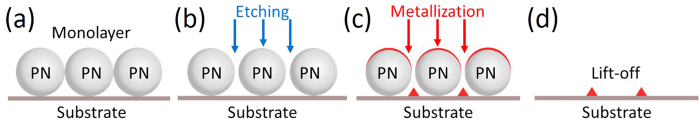
Steps of nanosphere lithography (**a**) monolayer formation (**b**) diameter reduction (**c**) metal deposition (**d**) lift-off.

**Figure 5:**
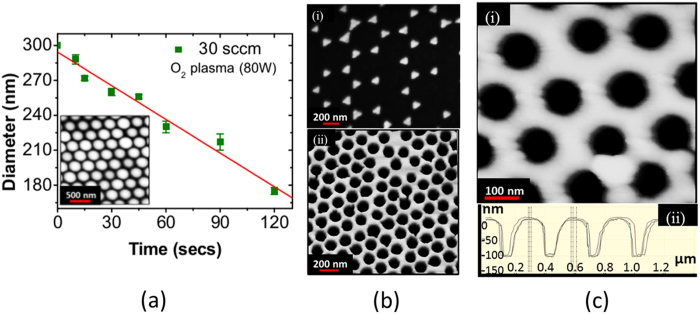
(**a**) Reduction in diameter of polystyrene nanospheres by oxygen plasma (RIE) as a function of etching time; inset:PNs etched for 45 secs. (**b**) SEM micrographs of nanometer features produced by NSL (i) array of Au nanodots (ii) chrome mask after lift-off (**c**) (i) AFM image of nanoholes on a Si/SiO_2_ substrate and (ii) their surface profile.

The monolayer produced was exploited as a mask to generate various patterns such as nanodots or nanoholes. To control the spacing (interstitial gaps) between the PNs, reactive ion etching (RIE) was used to reduce PNs diameter controllably (Fig. 4(a–d). This may be fine tuned by controlling the etch time while keeping the gas flow, pressure and power constant to create nanodots and nanoholes for short and long etch time, respectively. A short etch time (10 s) in oxygen plasma (30 sccm, 61 mTorr at 80 W) using Oxford Plasmalab 100 creates apertures almost 1/5^th^ the diameter of the nanospheres^25^ (Fig. 5a).

The deposition of a metal layer (~30 nm) in the interstitial gaps followed by the removal of the excess of PNs produces arrays of ~60 nm diameter nanodots (Fig. 5i) on Si (111) to serve as nucleation seeds for the growth of semiconductor III–V nanowires (data not shown). In a process labeled as lift-off, samples were immersed in toluene and sonicated for 2 mins to dissolve the polystyrene nanospheres, leaving behind the array of nanodots on the Si substrate. Longer etching is required to create nanoholes by reducing significantly the diameter of the nanospheres, as illustrated in Fig. 5a. A blanket deposition of a metal layer and the removal of the residual nanospheres leaves behind holes with a diameter similar to that of the etched nanospheres, i.e. 80–100 nm for 150 seconds RIE etching (Fig. 5ii). The surface profile of the bespoke sample was analyzed by Atomic Force Microscope (AFM) (Fig. 5ci,ii) showing the the holes to be smaller than 100 nm.

### Nanoimprint Lithography

**Figure 6:**
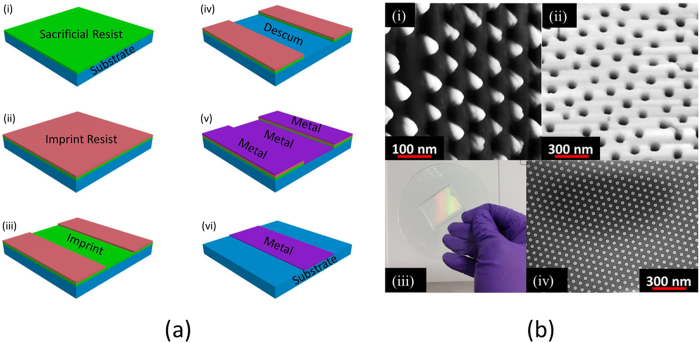
(**a**) Steps in bi-layer lift-off nanoimprint process: (i) spin coat sacrifical resist (ii) spin coat imprint resist (iii) stamp imprint under UV (iv) residual resist descummed (v) metallization (vi) lift-off (b) (i) nanodots produced by NSL and (ii) copied using PDMS (iii) photograph of PDMS stamp (iv) Au nanodots on Si.

Nanoimprint Lithography (NIL)^25^ is a lithographic technique based on the principle of direct mechanical deformation of the resist (Fig. 6a(i–vi)). It is based on replication, where the imprint resist is coated on a substrate before a high-resolution patterned stamp is pressed into the resist film by mechanical contact. The resist layer is UV or thermally cured under pressure. The residual layer is subsequently etched away before the patterned substrate undergoes further processing. We demonstrate that the samples patterned with nanodots (30–60 nm) or nanoholes (80–200 nm) by nanosphere lithography can be used as master stamps for NIL (Fig. 5bi, ii, iv). The quality of the stamp is critical to the resolution of the features produced. Various material properties such as hardness, thermal stability, thermal expansion coefficients, Poisson’s ratio, roughness, Young’s modulus are considered while selecting a mask. Hard stamps often use Si and SiO_2_ due to their process compatibility with both UV and thermal NIL while soft stamps usually exploit polydimethylsiloxane (PDMS) because of its replication properties. It is durable, inert to most materials being patterned or molded, and chemically resistant to most of the common solvents.

PDMS based composite stamp (5 × 4 cm) with nanodots (Fig. 6biii) were loaded in the imprint mask aligner, respectively. The recipe for the imprint based on the resist is setup for an optimum pressure (125 mbar) and exposure levels (dose: 1500 mJ/cm^2^). The process flow associated is illustrated in Fig. 6a(i–vi) while the pattern reproduced are shown in Fig. 6b(i, ii, iii). In essence, LOR 1A, a lift-off resist was spin coated at 3000 RPM for 1 min and baked at 200 °C for 7 mins. Amonil MMS4 (imprint resist) was spin coated at 3000 RPM for 1 min; no baking step is required. The stamp was loaded in the mask aligner and imprinted onto the resist at an optimum pressure and exposure. This led to the formation of the nanodots on the imprint resist. The residual layer left was plasma etched to expose the lift-off resist which was subsequently etched away from the open windows by immersing the sample in developer MF-319 for a few seconds. The desired metal(s) were deposited and the sample was immersed back in the developer MF-319 to etch off any remaining resist in order to expose the nanodots. The pattern reproducibility yield was shown to be as high as 95%, with a consistent replication of the features. The flexible stamp was used over 100 times with no sign of micro wear and tear. Figure 6b(iv) is a typical example of the reproducibility of the nandots via nanoimprint stamp produced by the bespoke method after 50 cycles, which exhibits an excellent resolution.

## Conclusion

We have demonstrated an efficient model for a spin-coating technique developed to create monolayers of polystyrene nanospheres on large-area substrates. The model exploits the interdependence of all the process parameters and forces involved to reduce the model to only two key convoluted parameters, the solid/liquid volume ratio and the spin speed. A phase plan describing the spin coating process was proposed. Unlike previous published work, we have demonstrated a better periodicity and surface coverage using nanospheres 300 nm in diameter instead of larger microspheres. This enabled us to produce even smaller features using the colloidal layer as a mask to create nanoscale patterns. By tailoring the etch time and oxygen flow, we can produce nanodots or nanoholes of varying sizes, 30–200 nm in diameter. These ‘as-fabricated’ structures can act as master templates, which were then copied using PDMS to produce flexible stamps for the production arrays of nanometer size feature by nanoimprint lithography. This offers an excellent potential to develop a low cost yet robust process.

## Methods

### Model boundary conditions

Given that liquid friction and viscosity are proportional to the spin speed, they cannot determine the direction of the process. The gravitational force for PNs with diameter 300 nm is nearly compensated by buoyancy. Centrifugal force is proportional to the square of rotation speed and becomes large enough to overcome gravity and roll over the PNs at 780 RPM for a 2 inch wafer. Roll over process cannot be taken into account below this speed. All the estimations and calculations necessary to support the model boundary conditions are detailed in the Supplementary Material section.

### Substrate preparation

2 inch silicon wafers (100) and (111), 300 *μ*m thick, 8–30 Ω. cm resistivity were used. For cleaning the wafers were immersed in piranha solution (H_2_SO_4_:H_2_O_2_ 3:1) for 10 mins, followed by a deionized (DI) water rinse. They were immersed in HF:H_2_O 1:10 for 1 min to remove the native oxide. They were thermally oxidized at 1040 °C to produce oxide thicknesses varying with oxidation times. The wafers were then cleaved into 1 × 1, 2 × 2, 3 × 3 cm^2^ small pieces while some wafers were left intact to test the scalability of the developed process.

### Solution formulation

PNs 300 nm in diameter suspended in ultrapure water (solid fraction of about 10%) were purchased from Sigma Aldrich. The PN suspension was diluted by varying the volume of triton X-100 and methanol (1:400), i.e. 1:1, 1:3, 1:5, 1:7. Viscosity measurements were conducted by a TA rheometer. The viscosity of water, methanol and triton X-100 (polyethylene glycol tert-octylphenyl ether) at 25 °C is 0.89, 0.54 and 240 mPa, respectively^26^,^27^. Given triton X-100 has a high boiling point^27^ of 270 °C, the evaporation of the solvent increases the concentration of triton X-100 whereas initially the solid/liquid ratio (R) is small, of the order 0.1. The volume varies with the size of substrates, e.g. 10 and 300 *μ*l for a 1 × 1 m^2^ substrate and for a full 2 inch wafer, respectively.

### Spin coating and characterisation

A Suss Delta 80 was used to spin coat the solution of polystyrene nanospheres on the respective samples at speeds from 50 to 10,000 RPM. The monolayers deposited were investigated by Hitachi s2400 scanning electron microscope (SEM), Veeco NanoMan atomic force microscope (AFM) and FEI Helios Nanolab focussed ion beam microscope. The scan spots were widely distributed across the wafer. Monolayer coverage is defined as the ratio of the monolayer area to the entire covered area whereas uniformity accounts for the dense-packing of the spheres. Oxford plasmalab reactive ion etching and an Edwards 306 thermal evaporator were used to etch the polystyrene and to deposit gold and chrome, respectively. Lift-off was carried out by sonicating the sample for 2 mins in toluene.

### Stamp production

A soft PDMS cushion layer and a hard PDMS (h-PDMS) imprint layer were designed. Soft PDMS was prepared by adding 10 parts of base (184 silicone elastomer) to 1 part of the initiator. 3.4 gm of Vinyl PDMS prepolymer, 18 ml of Pt catalyst, one drop of 2,4,6,8 tetramethylcyclotetrasiloxane and 1 ml of hydrosilane prepolymer were mixed together to create h-PDMS. The h-PDMS mixture was poured on the master stamp, spin-coated, then cured at 60 °C for 30 minutes prior to pouring PDMS mixture and curing for an hour at 75 °C. The mixture was subsequently poured gently on the master stamp pre-treated with CF_*x*_ plasma to render the surface hydrophobic. Finally, the PDMS sample was cured in a vacuum oven at 70 °C for an hour, after which, the cured PDMS is cooled down before it is gently pulled out from the master stamp.It is important to note that when the pattern on the master stamp is copied using PDMS, it gets inverted therefore in order to retain the same pattern, the bespoke PDMS stamp can be copied again using PDMS.

## Additional Information

**How to cite this article**: Chandramohan, A. *et al*. Model for large-area monolayer coverage of polystyrene nanospheres by spin coating. *Sci. Rep.* **6**, 40888; doi: 10.1038/srep40888 (2016).

**Publisher's note:** Springer Nature remains neutral with regard to jurisdictional claims in published maps and institutional affiliations.

## Supplementary Material

Supplementary Information

## Figures and Tables

**Table 1:** Effect of sphere packing on R and packing density at zero curvature of the hull.

Packing	Packing Density	Solid/liquid volume ratio (R)
Closed packed spheres	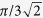	2.85
Cubic lattice in volume, square packing one layer	*π*/ 6	1.1
Hexagonal lattice in volume or one layer	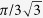	1.53
Lowest density rigid configuration in volume	0.494	0.975

**Table 2:** Various stages in proposed spin recipe and their effects on the final outcome (italics represents acceleration).

Stage	RPM	Duration	Phase	Dominant Force	Effect
A.1	150	120		Gravity	Solution spreads over substrate
*A.2*	*200*/*s*	—			
A.3	250	120	Spin-Up		Coverage improvement
*A.4*	*200*/*s*	—			
B.1	800	60			Partial coverage of disordered monolayer
*B.2*	*200*/*s*	—	Spin-Off	Centrifugal Force	
C.1	2500	20			Solvent volume reduces causing PNs to adhere to substrate
*C.2*	*200/s*	—			
C.3	5000	20	Self-Ordering		Hexagonal packing
*D.1*	*1000*/*s*	—		Surface Tension	
D.2	8000	360	Drying		Monolayer with hexagonal packing enhanced
