# Supplementary material for: Model for large-area monolayer coverage of polystyrene nanospheres by spin coating
Source: Sci Rep. 2017 Jan 19;7:40888. doi: 10.1038/srep40888 (PMC5244356; doi:10.1038/srep40888)
Supplement: Supplementary Information [file srep40888-s1.pdf]

Model for large-area monolayer coverage of polystyrene nanospheres by spin coating

Abhishek Chandramohan[1,*], Nikolai V. Sibirev[2], Vladimir G. Dubrovskii[2,3,4], Michael C. Petty[1], Andrew J. Gallant[1,4], and Dagou A. Zeze[1,4]

[1]Durham University, School of Engineering and Computing Sciences, Durham, DH1 3LE, United Kingdom
[2]St. Petersburg Academic University, St. Petersburg, 194021, Russia
[3]Ioffe Physical Technical Institute of the Russian Academy of Sciences, St. Petersburg, 194021, Russia
[4]ITMO University, St. Petersburg, 197101, Russia
[*]abhishek.chandramohan@durham.ac.uk

Supplementary Information

Model Boundary Conditions

In the spin recipe proposed, illustrated in Supplementary Fig. 1, the main forces acting during different stages of the process are gravity, inertia, surface tension and friction. Friction can be dry and liquid depending on the amount of solvent present on the substrate at a particular time during the process. For a low spin speed inertia is small and dry friction is less prominent than the gravitational force, allowing the latter to play an important role. However, the amount of solvent is enough to lubricate polystyrene spheres (PNs) which makes dry friction negligible. In most cases, for small rotation speeds, PNs float in the solvent. The gravitational force for PNs with diameter 300 nm is 1.8×10^{-21} N and is nearly compensated by buoyancy. Centrifugal force is proportional to the square of rotation speed and becomes large enough to overcome gravity and roll over the sphere at 780 rotations per minute for a 2 inch wafer.

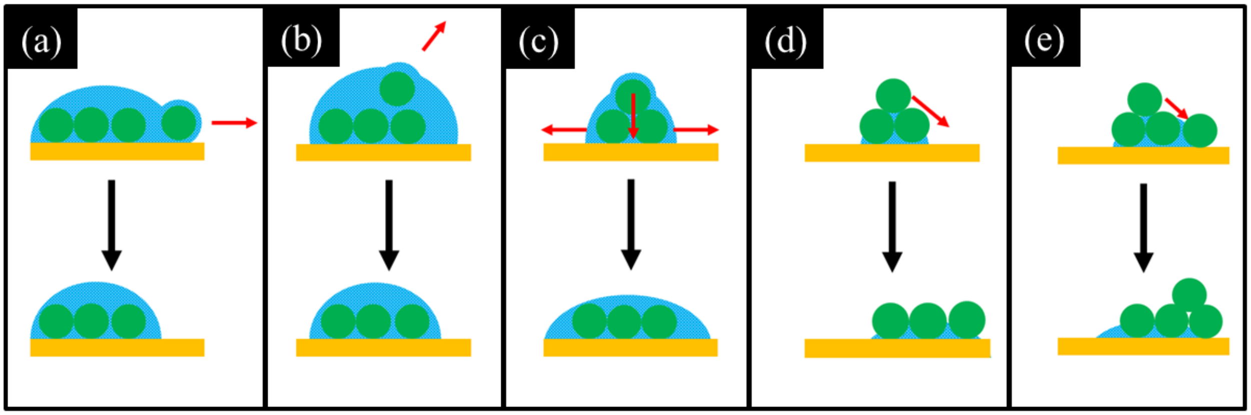

Figure 1. (Supplementary) Possible spreading routes of PNs during the spin coating process (a) sphere close to the edge escapes (b) loosely sitting sphere flung out (c) gravity assisted squeeze in (d) roll over to occupy the void (e) roll over on top of spheres

Roll over process cannot be taken into account below this speed. For instance, using surface tension of 20 mNm^{-1} and considering that the triple line length is $\sim$ 20 nm, the surface force is estimated at 2.5×10^{-9} N which makes it a million times greater than inertia and gravity. Therefore, when the curvature of the liquid becomes negative, surface tension becomes dominant. The curvature of the liquid surface is determined by the volume of the liquid (Supplementary Fig. 2a). Due to the hexagonal packing (Fig. 3g in main text) of PNs despite being spherical, a basic geometrical consideration (Supplementary Fig. 2b) is taken into account, making it easier to demonstrate that the curvature (Supplementary Fig. 2a) turns negative when the

solid/liquid volume ratio (R) becomes greater than 3/2 as shown below in supplementary eq. 1.

$$R > \frac{4\pi/3}{4\sqrt{3} - 4\pi/3} \approx \frac{3}{2} \tag{1}$$

The minimum amount of solvent required for the formation of a convex hull imposes an array of closed packed spheres. In this case, the space occupied by PNs is $\pi/\ 3\sqrt{2} \approx 0.74$. Hence, the critical radius (R_c) is about 2.85, when surface tension overcomes other forces. Beyond this point, rotational forces cannot destroy heaps of PNs. For an in plane close-packed spheres (hexagonal lattice), R is estimated differently as $\pi/(3\sqrt{3}\text{-}\pi) \approx 1.53$ whereas for the lowest density configuration of rigid packing of spheres the R is 0.97. For a monolayer of spheres with a square symmetry, a similar estimation gives R $\approx$ 1.1.

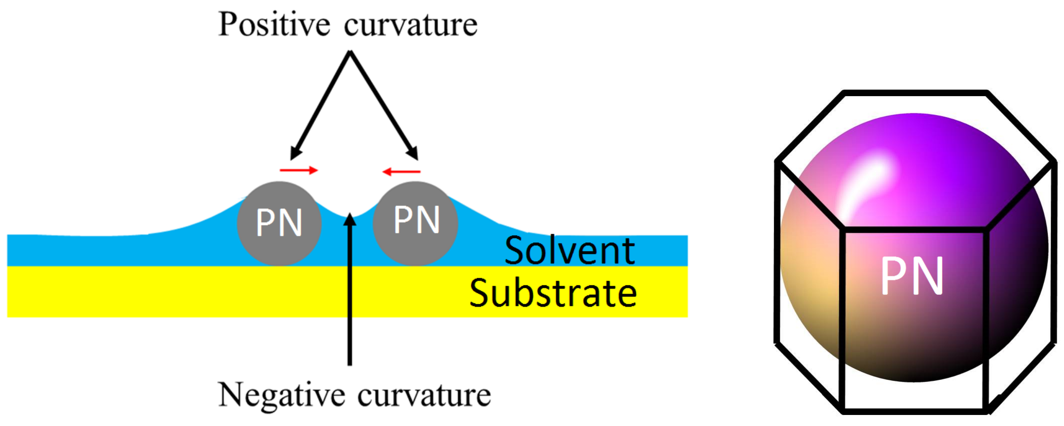

(a) (b)

Figure 2. **(Supplementary)** (a) Lateral capillary force acts when colloidal spheres are partially immersed in a thin layer of solvent (b) hexagonal lattice geometric consideration for the PNs

Surface tension always tightens the spheres inside the droplet. Since the concentration of triton X - 100 is more than 0.06%, the surface energy of the solvent is between 30 and 35 mNm^{-1}. The resulting gluing force is proportional to the mod of negative curvature of liquid surface. The force with which a liquid surface exerts on the PNs is proportional to the length of the triple contact line nanosphere-solvent-vapor and is routed into the liquid, see Fig. 1b (main text). The surface tension of an aqueous alcohol mixture can be varied from 20 to 73 mNm^{-1} due to the individual surface tensions of methanol and water[1,2] while the surface tension of the triton X-100 (surfactant) is 30 mNm^{-1}. Therefore, even for a very small distortion of solvent surface by a neighbouring nanosphere, surface tension overcomes all other surface forces and pulls the PNs together until they touch each other. This ensures rigid PNs packing or PNs fully covered by the solvent. Surface energy changes when the volume fraction of triton X - 100 changes from 0 to 3×10^{-6} mol m^{-2} as the concentration of methanol decreases from 25% to zero[3].

Solution formulation

Viscosity, which effectively determines the amount of liquid remaining on the substrate, strongly affects the volume ratio and surface tension[4,5]. Usually, the remaining liquid volume is proportional to the power law of the viscosity (supplementary eq. 2). Hence, an increase in the viscosity forces the phase space pathway downwards to the slow evaporation curve where the centrifugal force dominates[6].

$$h \sim \frac{\beta^B}{\omega^A} \tag{2}$$

where h is the thickness of the layer, ω is the rotation speed, β is the viscosity of the solution whereas A and B are process dependent factors. 300 nm diameter PNs suspended in ultrapure water (solid fraction of about 10%) were purchased from Sigma Aldrich. To control the viscosity, the suspension was diluted by adding varying ratios of a solution of triton X-100 and methanol (1:400), i.e. 1:1, 1:3, 1:5, 1:7. Viscosity measurements were conducted using a TA rheometer. The viscosity of water, methanol and triton X-100 at 25 °C is 0.89, 0.54 and 240 mPa, respectively[7,8]. The viscosity of our solution[7] is higher than that of individual components, e.g. the viscosity of 40% methanol in water solution is 1.59 mPa (Supplementary Fig. 3). Given triton X-100 has a high boiling point[8] of 270°C, the evaporation of the solvent increases the concentration of triton X-100

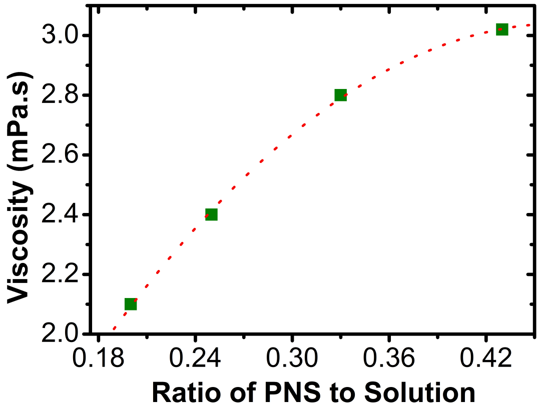

Figure 3. **(Supplementary)** Change in viscosity of the solution w.r.t ratio of PNS to the solution

whereas initially, the solid/liquid ratio (R) is small, of the order 0.1. However, as self-ordering proceeds, the ratio R must be between 1 and 10 which is inline with table 1 (main text). A one or two orders of magnitude increase in the concentration of triton X–100 causes the viscosity to rise. Methanol rapidly evaporates in the initial stage, leading to a change in the viscosity and surface tension. Thus, to reduce the detrimental effects of high viscosity on the initial stage, triton X-100 was diluted with methanol. This procedure is optimized for the best concentration of triton X-100 in the final solution. In fact, triton X–100 drastically changes the viscosity of the solvent and stabilizes the surface tension. The composition and volume of the solution are directly related to the number of PNs present in the initial solution, hence, play a major role in the formation of a PN monolayer. The volume varies with the size of the substrates, e.g. 10 and 300 μl for a $1 \times 1\text{cm}^2$ substrate and for a full 2 inch wafer, respectively. Minimizing the volume of solution for large area substrates imposes that a balance is found in relation with spin speed. Differential weight measurement before and after spin coating was carried out to determine the number of spheres lost during the entire process and to identify the appropriate amount of the solution required for the process. For instance, the numbers of spheres in a 50 μL droplet was estimated at $\approx 3 \times 10^{15}$. Residual weight measurement after spin coating showed that the number of PNs left on the substrate was $\approx 5 \times 10^{14}$, an order of magnitude smaller.

References

1. Aldrich, S. Triton x-100. *URL accessed July 2016* **MSDS**, 1907 (2016).
2. Vazquez, G., Alvarez, G. & Navaza, J. M. Surface tension of alcohol and water from 20 to 50°c. *J. Chem. Eng. Data* **40**, 611–614 (1995).
3. Zdziennickam, A. The adsorption properties of short chain alcohols and triton x-100 mixtures at the water–air interface. *J. Colloid and Interface Science* **335**, 175–182 (2009).
4. Meyerhofer, D. Characteristics of resist films produced by spinning. *Journal of Applied Physics* **49**, 3993 (1978).
5. Sahu, N., Parija, B. & Panigrahi, S. Fundamental understanding and modeling of spin coating process: A review. *Indian J. Phys.* **83(4)**, 493–502 (2009).
6. Mikhail, S. & Kimel, W. Densities and viscosities of methanol-water mixtures. *Journal of chemical and engineering data* **6(4)**, 533 (1961).
7. Bornstein, L. Properties of matter in its aggregated states. *Viscosity and diffusion* **II**, 5a (1969).
8. Aldrich, S. http://www.sigmaaldrich.com/catalog/product/sial/x100?lang=en&region=GB July (2016).
